# Requirement of Smad4 from Ocular Surface Ectoderm for Retinal Development

**DOI:** 10.1371/journal.pone.0159639

**Published:** 2016-08-05

**Authors:** Jing Li, Shusheng Wang, Chastain Anderson, Fangkun Zhao, Yu Qin, Di Wu, Xinwei Wu, Jia Liu, Xuefei He, Jiangyue Zhao, Jinsong Zhang

**Affiliations:** 1 Department of Ophthalmology, the Fourth Affiliated Hospital of China Medical University, Eye Hospital of China Medical University, Key Lens Research Laboratory of Liaoning Province, Shenyang, Liaoning Province, China; 2 Department of Cell & Molecular Biology, Tulane University, New Orleans, Louisiana, United States of America; 3 Department of Ophthalmology, Tulane University, New Orleans, Louisiana, United States of America; 4 Shenyang Fourth People's Hospital, Shenyang Eye Research Institute, Shenyang, Liaoning Province, China; 5 Department of Ophthalmology, Ningbo No.2 Hospital, Ningbo, Zhejiang Province, China; National Eye Centre, UNITED STATES

## Abstract

Microphthalmia is characterized by abnormally small eyes and usually retinal dysplasia, accounting for up to 11% of the blindness in children. Right now there is no effective treatment for the disease, and the underlying mechanisms, especially how retinal dysplasia develops from microphthalmia and whether it depends on the signals from lens ectoderm are still unclear. Mutations in genes of the TGF-β superfamily have been noted in patients with microphthalmia. Using conditional knockout mice, here we address the question that whether ocular surface ectoderm-derived *Smad4* modulates retinal development. We found that loss of *Smad4* specifically on surface lens ectoderm leads to microphthalmia and dysplasia of retina. Retinal dysplasia in the knockout mice is caused by the delayed or failed differentiation and apoptosis of retinal cells. Microarray analyses revealed that members of Hedgehog and Wnt signaling pathways are affected in the knockout retinas, suggesting that ocular surface ectoderm-derived *Smad4* can regulate Hedgehog and Wnt signaling in the retina. Our studies suggest that defective of ocular surface ectoderm may affect retinal development.

## Introduction

Microphthalmia, a common congenital ocular disease, is characterized by abormal small eyes with leukoma, cataract, aniridia, anterior and posterior synechiae, retinal detachment, retinal folds and so on. The estimated birth prevalence of this condition is 1 per 7000, but it was reported in 3.2–11.2% of blind children [1, 2]. Genetic causes account for approximately 80 percent of the disease [1]. However, the underlying cellular and molecular mechanism of microphthalmia pathogenesis remains unclear. To date, the abnormality in the anterior segment of microphthalmia, such as leukoma, cataract and aniridia, can be relieved by cataract phacoemulsification combined intraocular lens implantation, keratoplasty and other surgical treatments. However, there is no effective treatment to alleviate or prevent the pathological retinal changes that underlie the vision impairment in the disease. Part of the reasons include: the pathological changes of the retinas in the disease is not well-defined, and the mechanisms where by defects in lens impact retinal development is still unclear.

Interaction between neuroepithelium and ectoderm plays an essential role in vertebrate eye development. It is well established that lens development requires induction from the protruding optic vesicle, while optic vesicle invagination and retinal maintenance demand lens-to-retina signaling [3–9]. Numerous signaling pathways, including Sonic hedgehog (Shh), fibroblast growth factor (FGF), transforming growth factor-β (TGF-β), bone morphogenetic protein (BMP) and Wnt signaling pathways [10–12], are required for eye development. However, how these signaling pathways regulate the interaction between lens and retina especially in pathological conditions in microphthalmia remains unclear. Mutations in genes of the TGF-β superfamily have been found in patients with microphthalmia [13]. *Smad4* is a key intracellular effector of TGF-β superfamily of secreted ligands. Previous research has shown that deletion of *Smad4* in the lens ectoderm of mice leads to severe abnormality in the anterior segment and microphthalmia [14]. However, whether lens ectoderm-derived *Smad4* is required for retinal development is still unknown. Here we present data that *Smad4* in the ocular surface ectoderm is required for retinal development. Retinal dysplasia was observed in mice when *Smad4* is knocked out only in the ocular surface ectoderm. This phenotype likely results from abnormal differentiation and apoptosis of retinal cells. Mechanistically, *Smad4* in the lens ectoderm affects Shh and Wnt signaling in the retina.

## Materials and Methods

### Animals

All animal experiments followed the guidelines of the Association for Research in Vision and Ophthalmology Statement for the Use of Animals in Ophthalmic and Vision Research and were approved by the Animal Use Committee of the Institute of Zoology, Chinese Academy of Sciences (approval number IOZ150108). Le-Cre transgenic mice [15], *Smad4*^flox/flox^ mice [16] and ROSA26 reporter mice [17] were kindly gifted from Dr. Yi-Hsin Liu (University of Southern California, Los Angeles). All mice were kept under specific pathogen free (SPF) conditions at 20–26°C, 40–70% humidity with a 12 hour light/dark cycle, in ventilated polycarbonate mouse cages (32 x 20 x 18 cm), and had free access to standard chow and drinking water. The Le-Cre heterozygousmice were bred with mice carrying floxed *Smad4* alleles (*Smad4*^flox/flox^). The male mice carrying Le-Cre;*Smad4*^flox/+^ were subsequently crossed with females carrying *Smad4*^flox/flox^ to generate Le-Cre;*Smad4*^flox/flox^ (*Smad4*-cKO) mice. Littermate mice carrying *Smad4*^flox/flox^ were used as controls. PCR was performed to detect the genotype of mice with DNA samples extracted from the tail or embryo yolk sac. The specific primer sets were used as previous described [15–17].

### GFP fluorescence and LacZ staining

In the mice carrying Le-Cre, the first Pax6 promoter (P0) was cloned upstream of sequences encoded the nls-Cre followed by internal ribosome binding sites (IRES) and green fluorescent protein (GFP) [15]. GFP fluorescence was detected in Le-Cre eye sections at E11.5 to show the location of Cre. The ROSA26R mouse strain was used for monitoring Cre expressing cells, by crossing with Le-Cre expressing strains [17]. Embryos of the Le-Cre;ROSA26 were used for Lacz staining, which was performed as previously described [17].

### Hematoxylin and Eosin staining

Mice were anesthetized by sevoflurane and then sacrificed by cervical dislocation to get embryos or eyeballs, and efforts were made to minimize the number of animals used and their suffering. Embryos or postnatal eyes were dissected and fixed in 4% paraformaldehyde (embryos) or Davidson’s fixative (postnatal eyes) [18] overnight at 4°C. The samples then underwent graded alcohols dehydration, clearing in xylene and embedded in paraffin. 4μm sections were cut for using. Sections were stained with Hematoxylin and Eosin and pictures were taken using an Olympus light microscope equipped with a Spot CCD camera.

### Retinal thickness measurement and cell counting

Nine retinal sections were employed in each item measurement or counting, obtained from three independent animals of different litters. The group of nine sections from retinas of each age was collected from the central area of the eye balls through the optic nerve.

The retinal thickness at different points was measured using Image-Pro Plus 6.3, including nasal central zone, nasal paracentral zone, nasal peripheral zone, temporal central zone, temporal paracentral zone, temporal peripheral zone. In central areas, the retinal thickness was measured at 50μm (embryos) or 200μm (postnatal eyes) eccentric from the optic nerve head site, and the measurement at 50μm (embryos) or 200μm (postnatal eyes) eccentric from the anterior margin of retina was regarded as the peripheral zone. The retinal thickness of paracentral zone was measured at the midpoint of the unilateral retina. Mean thickness at each point was calculated on sections of each age.

For total positive cell counting of one section, the whole retina was partial captured in several ×400 areas, then compositing these pictures into a integrated one. The number of positive cells was counted in each area and then calculated the total number in the whole retina. The total number of retinal cells was counted in sections with nucleus staining by Hematoxylin or DAPI. Mean number of each kind of positive cell was calculated on sections of each age. Regional positive cell counting was also performed in a 10^−2^ mm^2^ (embryos) or 10^−1^ mm (postnatal eyes) area at nasal central zone, nasal paracentral zone, nasal peripheral zone, temporal central zone, temporal paracentral zone and temporal peripheral zone, respectively. Mean number at each area was calculated.

### Immunostaining

The paraffin sections underwent deparaffinization and rehydration. Epitope retrieval was performed in 0.1M sodium citrate buffer (pH 6.0) at 100°C for 10 minutes, then blocking for 1h with 5% BSA. After the addition of primary antibodies, sections were incubated in a humidified chamber at 4°C overnight. After three washing steps with PBS, the secondary antibodies were added and incubation continued at room temperature for 1 hour. Cell nuclei were counterstained with DAPI and pictures were taken using an Olympus fluorescence microscope equipped with a Spot CCD camera. The primary and secondary antibodies are listed in S1 Table.

### BrDU labeling and TUNEL assay

BrDU (100 mg/g body weight) (B-5002; Sigma-Aldrich, St. Louis, MO) was injected intraperitonealy in pregnant mice for 1 hour or post-natal mice for 2 hours before sacrifice [19]. Immunostaining was performed as described above. Apoptotic cells were detected by employing the Fluorescein In situ Cell Death Detection Kit (TMR red, Roche, Basel, Switzerland). Briefly: deparaffinized sections were treated with Proteinase K (20 μg/ml) for 20 min, fragmented DNA was labeled with fluorescein-dUTP using terminal transferase, cell nuclei were counterstained with DAPI, and slides were mounted with anti-fade medium.

### Microarrays and Quantitative PCR of Retinal Tissue

RNA was extracted from retina isolated from control and *Smad4*-cKO mice at P0 using RNeasy micro kit (Cat#74004, QIAGEN, GmBH, Germany). The microarrays were carried out by China National Engineering Center for Biochip at Shanghai using One-Color Microarray-Based Gene Expression Analysis, and subsequent analyses conducted using SAS statistical software online (http://sas.ebioservice.com/). Quantitative PCR (qPCR) was carried out to verify the microarrays results using SYBR Premix Ex TaqTM II (Takara, Dalian, China) and analyzed based on the equation RQ = 2^−ΔΔCT^. The sequences of real-time qPCR primers are listed in S2 Table.

### In Situ Hybridization

Embryos or postnatal eyes were dissected and fixed in 4% paraformaldehyde overnight at 4°C. The samples then underwent 15% and 30% sucrose dehydration, and embedded in OCT compound (Sakura Finetek, Torrance, CA). 10μm sections were cut for using. In situ hybridization was performed as previously described [20, 21]. The Gli2, Gli3 and Wnt2b cDNA, obtained by above real-time qPCR, underwent PCR with Sp6-Gli2-T7, Sp6-Gli3-T7 and Sp6-Wnt2b-T7 primers listed in S2 Table, to get the Gli2, Gli3 and Wnt2b cDNA with T7 and Sp6 promoters. The cDNA was purified using SanPrep Column PCR Product Purification Kit (Sangon Biotech, Shanghai, China). cDNA sequencing was also performed by Sangon Biotech to verify the base sequences.

### Statistical analyses

Statistical evaluations between control and *Smad4*-cKO samples were performed using the unpaired Student’s t-test (two-tailed).

## Results

### *Smad4* deletion in the ocular surface ectoderm results in microphthalmia, aphakia and hypoplasia in the ciliary body and iris

**Fig 1:**
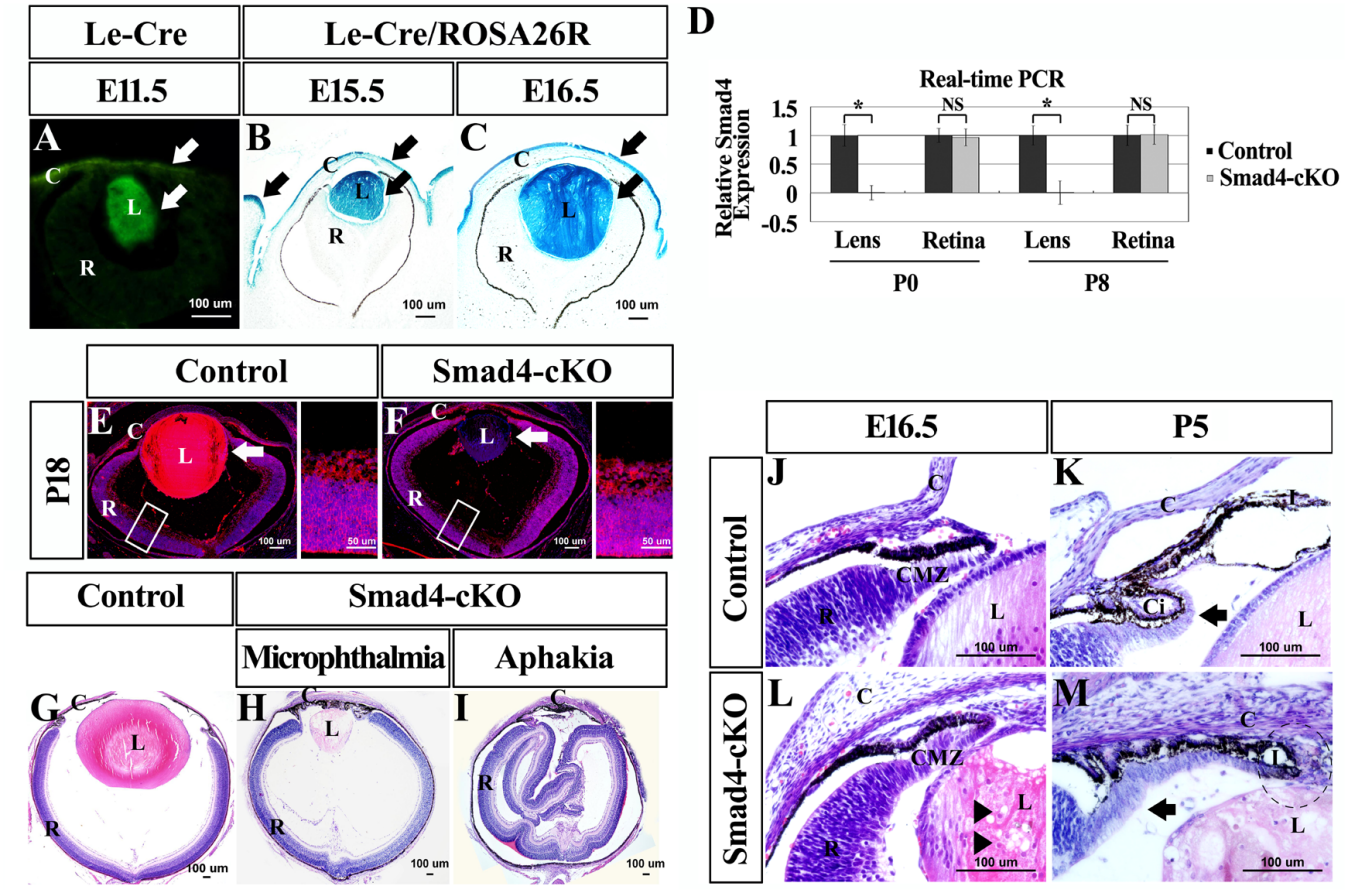
Varying degrees of microphthalmia or aphakia in Smad4-cKO mutants compared to the wild type mice. **(A)** The expression pattern of Cre recombinase was specifically observed in the lens and cornea by detecting GFP fluorescence (arrows). **(B-C)** By crossing Le-Cre mice to ROSA26 reporter mice, Cre-mediated recombination was specifically observed in the lens, cornea and eyelids (arrows). **(D)** Real-time qPCR was employed to detect the expression of *Smad4* in lens and retina at P0 and P8, respectively. n = 4, *P<0.05. **(E-F)** Immunostaining was performed to detect the expression of *Smad4* in cKO eyes of E18.5, which showed the specific loss of *Smad4* in the lens but not retina (arrows). **(G-I)** Microphthalmia and aphakia were observed in *Smad4*-cKO mutants at P7. In the aphakia mutant, the retina appeared pleated and corrugated in the center of the eye ball, while in microphthalmia, the retina formed a cup-structure and showed a proper orientation. **(J-M)** Abnormal development of the CMZ presented in the *Smad4*-cKO compared to the wild type mice at E16.5 and P5. Presence of cortical vacuoles in the mutant lens (arrowheads). At E16.5, the *Smad4*-cKO mice presented normal forward extension and early development of the ciliary body and iris as shown by thinning of the periphery of the retina (CMZ). But at P5, the iris stroma showed hypoplasia with the cornea, iris, and lens attached to each other (round frame). Furthermore, the neuroepithelium failed to fold backward to form ciliary body (arrows). E, embryonic; P, postnatal; M, month; L, lens; C, cornea; R, retina; I, iris; Ci, ciliary body; CMZ, ciliary marginal zone.

To address whether *Smad4* in the surface ectoderm of the eye is required for retinal development, we generated conditional *Smad4* knockout mice (*Smad4*-cKO) using Le-Cre as deletor [15, 16]. In the mice carrying Le-Cre, the first Pax6 promoter (P0) was cloned upstream of sequences encoded the nls-Cre followed by internal ribosome binding sites (IRES) and green fluorescent protein (GFP) [15]. The ROSA26R mouse strain was of wide use for monitoring the lineage of Cre expressing cells. Crossing ROSA26R mice with Le-Cre expressing strains can activate the ROSA26 promoter and lacZ expression in cells where Cre expressed [17]. The expression pattern of Cre recombinase was specifically observed in the lens, cornea and eyelids, by detecting GFP that co-expresses with Cre and using the ROSA26-LacZ reporter mouse strain (Fig 1A–1C). The efficiency of *Smad4* knockout was also confirmed by the specific loss of *Smad4* in the lens but not retina (Fig 1D–1F). Consistent with a previous publication, microphthalmia was observed in the *Smad4*-cKO mice [14] (Fig 1G and 1H and, S1A–S1C Fig). Various degrees of microphthalmia with atresia iridis (appeared in 90% of the cases) or even aphakia (appeared in 10% of the cases) was observed in the *Smad4*-cKO mutant (Fig 1G–1I and S1A–S1C Fig). In the cKO mice with aphakia, the retina appeared pleated and corrugated in the center of the eye ball; while in mutant with microphthalmia, the retina formed a cup-structure and showed a proper orientation (Fig 1H and 1I). At embryonic (E) day 10.5, in both wildtype (WT) and cKO mice, the optic vesicle and the thickened surface ectoderm invaginated together to form the optic cup, and no difference in the size of the optic cup was observed (S2A and S2E Fig). At E12.5, the mutant eye was slightly smaller, then the volume of the mutant eye became significantly smaller compared to the WT controls as the embryos developed, as indicated by the decreased length of both antero-posterior diameter and left-right diameter. (S2B–S2D, S2F–S2H and S2J Fig). Moreover, the cKO mice showed smaller lens and congenital cataracts (Fig 1G and 1H), as shown by histology and the vacuolation of the lens nucleus (Arrowheads, Fig 1L), and dysplastic primary vitreous accompanied by large numbers of vessels and nucleated erythrocytes attached to the posterior lens capsule (Arrows, S2F and S2G Fig).

Most of the iris and ciliary body cell types originate from peripheral progenitors of the retina. *Smad4*-cKO mice also showed defective ciliary body and iris formation, which has not been documented before. At E16.5, the *Smad4*-cKO mice presented normal forward extension and early development of the ciliary body and iris as shown by thinning of the periphery of the retina (Fig 1J and 1L). However, at P5, the iris stroma in the cKO mice showed hypoplasia with the cornea, iris, and lens attached to each other (Fig 1M). Furthermore, the neuroepithelium in the cKO mice failed to fold backward to form ciliary body (Fig 1K and 1M). Taken together, deletion of *Smad4* in the ocular surface ectoderm leads to microphthalmia, aphakia and hypoplasia of ciliary body and iris.

### *Smad4* deletion in surface ectoderm leads to defective retinal development

**Fig 2:**
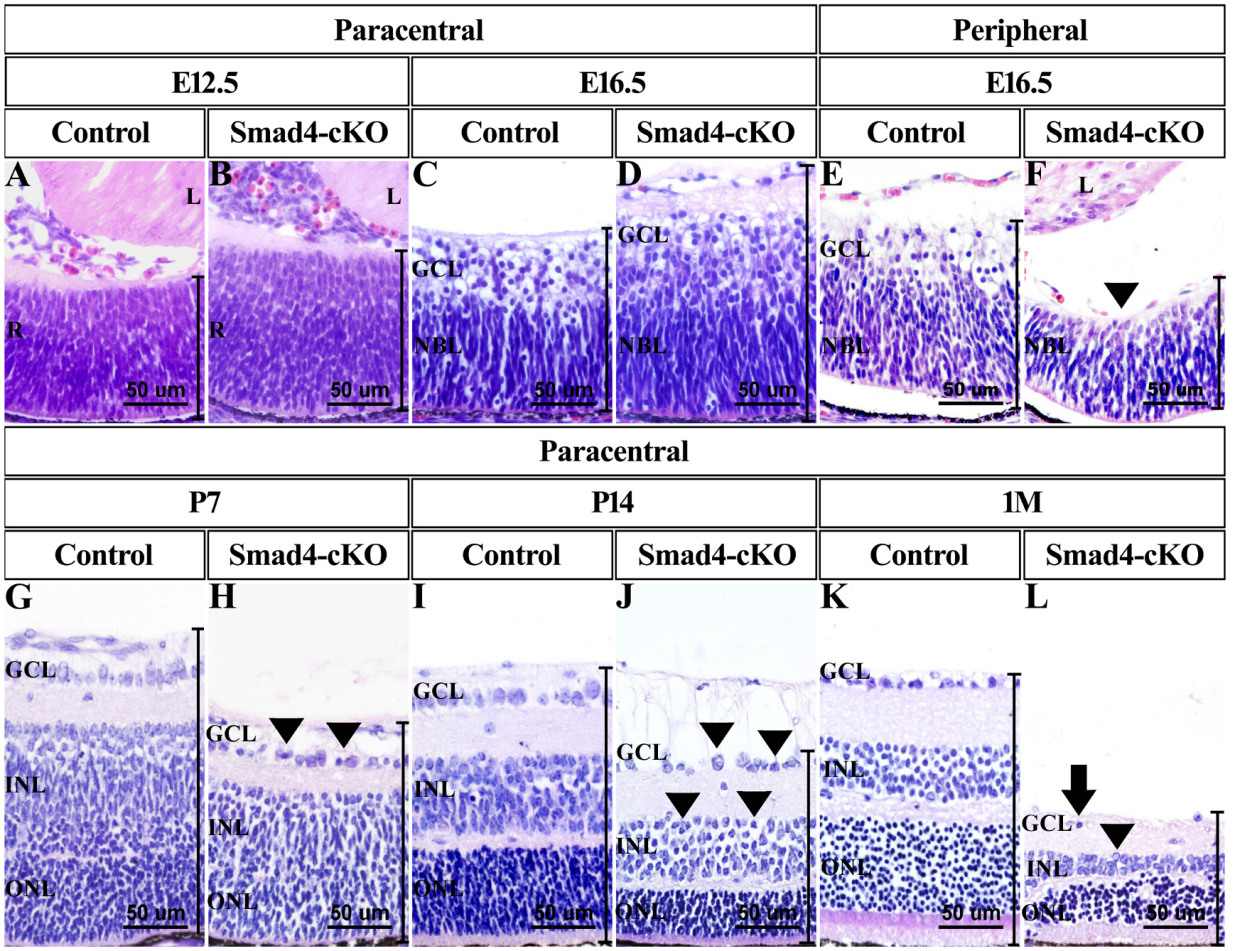
Deletion of *Smad4* led to changes of retina thickness and degeneration of retinal cells. **(A-D, G-L)** Photographs of retina showed the nasal paracentral zone of cKO mice and WT mice of the indicated stages. The retina of cKO mice appeared thicker compared to control retina at embryonic stages. After P3, the thickness of retina of *Smad4*-cKO mice reduced dramatically. The retinal cells of cKO mice were unevenly arranged and displayed highly variable shape and sizes (arrowheads). At 1 month, there were hardly any ganglion cells (arrow). **(E, F)** Photographs of the nasal peripheral zone of cKO and control mouse retina, at E16.5. Ganglion cell layer failed to arise (arrowheads). E, embryonic; P, postnatal; M, month; L, lens; R, retina; GCL, ganglion cell layer; NBL, neuroblastic layer; INL, inner nuclear layer; ONL, outer nuclear layer.

To test whether deletion of *Smad4* in ocular surface ectoderm has impact on retinal development, the morphology of the retinas in the cKO mice was examined. Surprisingly, retinas in the cKO mice appeared thicker compared to the WT mice from E12.5 to postnatal (P) day 3 (Fig 2A–2D, S3A Fig). Particularly, the thickness of ganglion cell layer and neuroblast layer are more pronounced at both the central zone and the paracentral zone before birth in the cKO mice compared to the WT controls (S3C and S3D Fig). However, the ganglion cells layer was not present at peripheral zone at E16.5 in the cKO mice (Fig 2E and 2F, S3C Fig). The total number of retinal cells in the *Smad4*-cKO mice was significantly more than that in the WT controls, although the cellular morphology appeared normal (Fig 2A–2D, S3B Fig). However, after P3, the thickness of the cKO retina reduced dramatically and uniformly at central zone, paracentral zone, peripheral zone, due to decreased thickness in each layer (Fig 2G–2L, S3A, S3C–S3F Fig). Consistently, the total number of retinal cells in cKO mice declined sharply (S3B Fig). Especially, the ganglion cell layer and inner nuclear layer (or inner layers of neuroblast layer) were unevenly arranged and displayed highly variable shapes and sizes after P3 (Arrowheads, Fig 2H, 2J, and 2L). The abnormality first appeared in the ganglion cell layer, and subsequently the cells in inner nuclear layer (Arrowheads, Fig 2H, 2J, and 2L). Retinal folds and retinal detachment were frequently observed in mutant retina after P7 (S2D, S2H, and S2I Fig). The nuclei were polarized away from a “lumen” to form a rosette structure (*, S2I Fig). At 1 month, there were hardly any ganglion cells detected in *Smad4*-CKO retina (Fig 2L). These results indicate an initial overgrowth of the retinal cells followed by retinal degeneration when *Smad4* is deleted in the ocular surface ectoderm.

### Conditional deletion of *Smad4* on surface ectoderm affects cell proliferation and apoptosis in the retina

**Fig 3:**
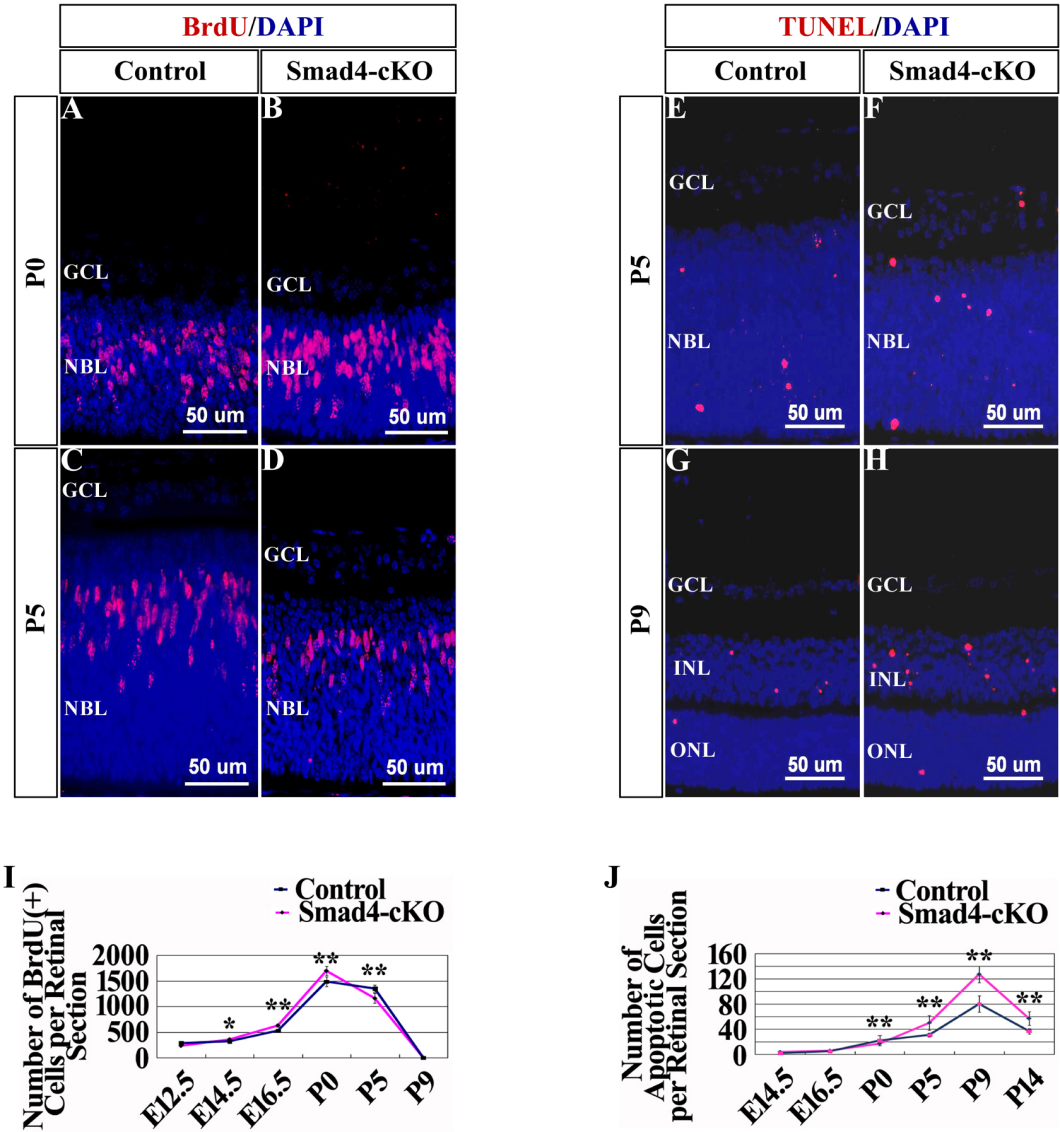
Loss of *Smad4* led to excessive proliferation of the retina cells at early development and abundant apoptosis later as assessed by pulse BrDU labeling and staining as well as TUNEL assay. **(A-D)** Results of BrDU labeling followed by immunostaining were shown in *Smad4*-cKO mice and control mice at the nasal paracentral zone of P0 and P5. **(E-H)** TUNEL assay results were shown in *Smad4*-cKO mice and control mice at the nasal paracentral zone of P5 and P9. The *Smad4*-cKO retina exhibited more apoptosis at P5 and P9. **(I)** Quantification of total BrdU^+^ cells per retinal section in *Smad4*-cKO mice and control mice. From E14.5, the number of BrdU^+^ cells in the retina of cKO mice was significantly more than that of control. After birth, the number of BrdU^+^ retinal cells started to decrease in mice, with more decrease observed in cKO mice than in controls. n = 9, *P<0.05; **P<0.01. **(J)** Quantification of total apoptosis cells per retinal section in *Smad4*-cKO mice and control mice. In the first week after birth active retinal remodeling occurred and retinal cell apoptosis was detected in control mice. However, the *Smad4*-deficient retina exhibited more apoptosis than the control. n = 9. *P<0.05; **P<0.01. E, embryonic; P, postnatal; M, month; GCL, ganglion cell layer; NBL, neuroblastic layer; INL, inner nuclear layer; ONL, outer nuclear layer.

To determine the cellular mechanism underlying the retinal phenotype in the *Smad4*-cKO mice, cell proliferation and cell death in the retina was measured. Cell proliferation was examined by pulse BrDU labeling and staining. At E12.5, there was no significant difference in the number of BrdU^+^ retinal cells between the *Smad4*-cKO and WT mice (Fig 3I). However, from E14.5 to newborn, there was significantly more increase in the number of BrdU^+^ cells in the cKO retina compared to those in the WT controls in the central, paracentral and peripheral zones (Fig 3A, 3B and 3I, S4B Fig). After birth, the number of BrdU^+^ retinal cells started to decrease in mice, with more decrease observed in cKO mice than in controls (Fig 3C, 3D and 3I). At P9, the BrdU^+^ cells were rare in both control and the *Smad4*-CKO retinas (Fig 3I). We also examined retinal cells death using TUNEL assay. At the embryonic stage, there were few apoptotic cells in the retina of both control and *Smad4*-CKO mice (Fig 3J). In the first week after birth active retinal remodeling occurred and retinal cell apoptosis was detected in WT control mice. *Smad4*-cKO retinas showed significantly more cell death than the controls in each layer, especially at peripheral zone after P0 (Fig 3E–3H and 3J, S4D Fig).

### *Smad4* deletion on surface ectoderm influences the differentiation of retinal cells

**Fig 4:**
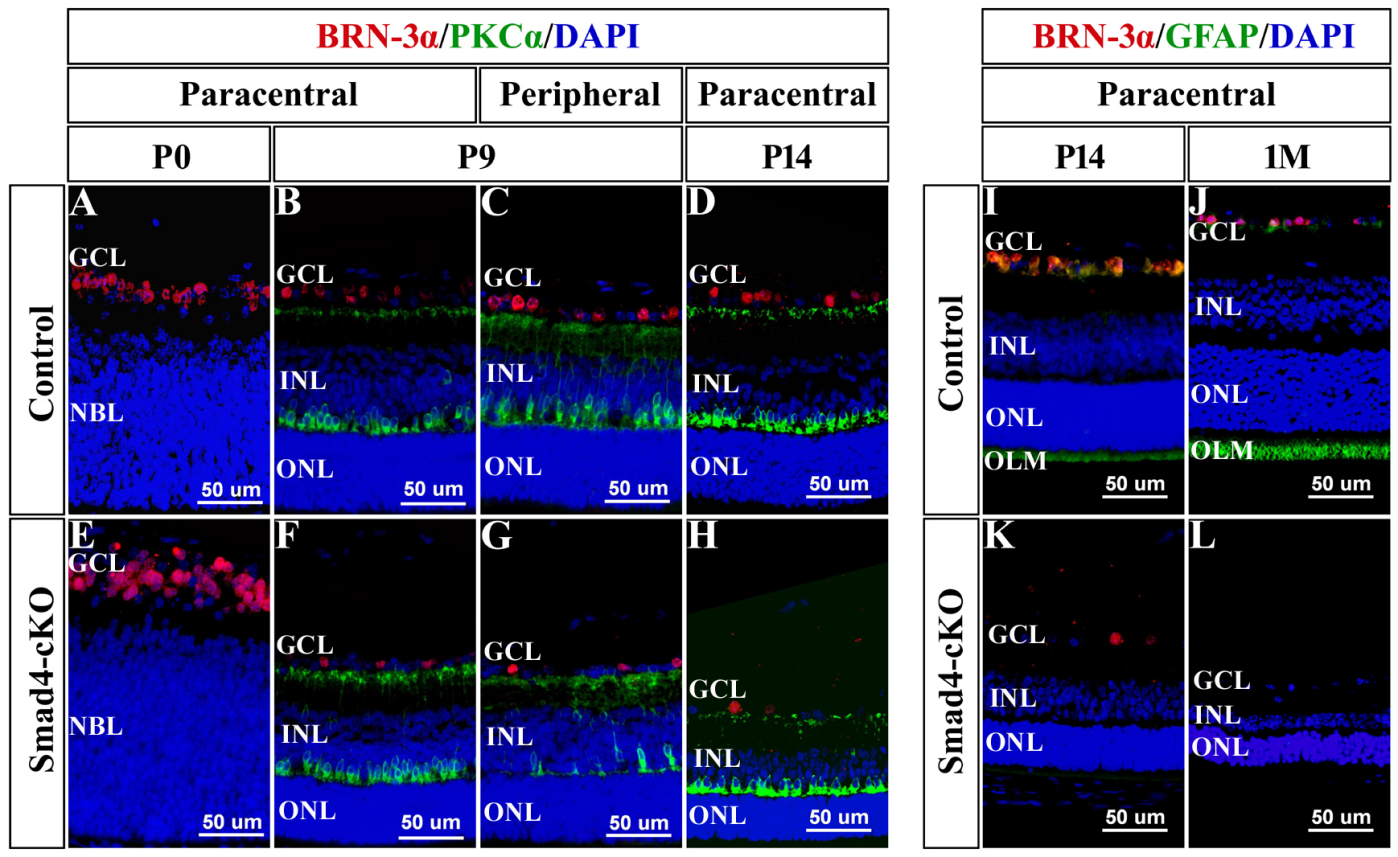
Loss of *Smad4* affected the differentiation of retinal ganglion cells, bipolar cells, and Müller cells. **(A-H)** Immunostaining was performed to label retinal ganglion cells (red) and bipolar cells (green) in P0, P9 and P14 *Smad4*-cKO and control mice. At P0, the number of ganglion cells in cKO retina was significantly more than that in control. At P9 and P14, the number of ganglion cells in cKO retina was significantly decreased. The bipolar cells showed delayed differentiation mainly at peripheral zone at P9. **(I-L)** Immunostaining was performed to label retinal ganglion cells (red) and Müller cells (green) in *Smad4*-cKO and control mice at P14 and 1M. In *Smad4*-cKO retina, the expression of GFAP was not observed from P9 through 1M after birth, indicating failed differentiation of Müller glia. E, embryonic; P, postnatal; M, month; GCL, ganglion cell layer; NBL, neuroblastic layer; INL, inner nuclear layer; ONL, outer nuclear layer; OLM, outer limiting membrane.

Since *Smad4*-cKO retinal cells showed morphological abnormality mainly in the ganglion cell layer and inner nuclear layer, we employed Brn-3α, PKCα and GFAP antibodies to investigate the differentiation of ganglion cells, bipolar cells, and Müller cells respectively. In the wild-type retina, the number of retinal ganglion cells presented a progressive increase across embryonic stages and a subsequent, substantial reduction due to retinal remodeling during the first postnatal week (Fig 4A–4D, 4I and 4J, S5B Fig). However, in the cKO mice, the Brn3α^+^ ganglion cells showed delayed differentiation at peripheral zone at E16.5 (S5A Fig), since the number of ganglion cells was significantly less than that of control at peripheral zone (S5D Fig). The total number of ganglion cells was apparently more than that of control across embryonic stages (S5B Fig). However, the Brn3α^+^ ganglion cells decreased after birth, with significantly more decrease detected in cKO mice (Fig 4F–4H and 4K and 4L, S5B Fig), which is consistent with our histological observation and could be explained by increased apoptosis. The retinal bipolar cells (labeled by PKCα) are differentiated from neuroblasts around P7, and the number of bipolar cells increased gradually afterward (Fig 4B–4D, S5C Fig). In the cKO retina, the bipolar cells showed delayed differentiation mainly at peripheral zone, and the total number of bipolar cells was obviously less at P9 (Fig 4F and 4G, S5C Fig). After P9, the number of cKO bipolar cells increased and showed no difference to control (Fig 4H, S5C Fig). At 1M after birth, PKCα^+^ bipolar cells decreased in the cKO retina probably caused by increased apoptosis (S5C Fig). Müller cells (labeled by GFAP) are generated around P9 in wild-type retina. However, GFAP expression was not observed in *Smad4*-cKO retina, indicating failed differentiation of Müller glia (Fig 4I–4L).

### Regulation of genes in Hh and Wnt pathways in the retina by *Smad4* deletion in the ocular surface ectoderm

**Fig 5:**
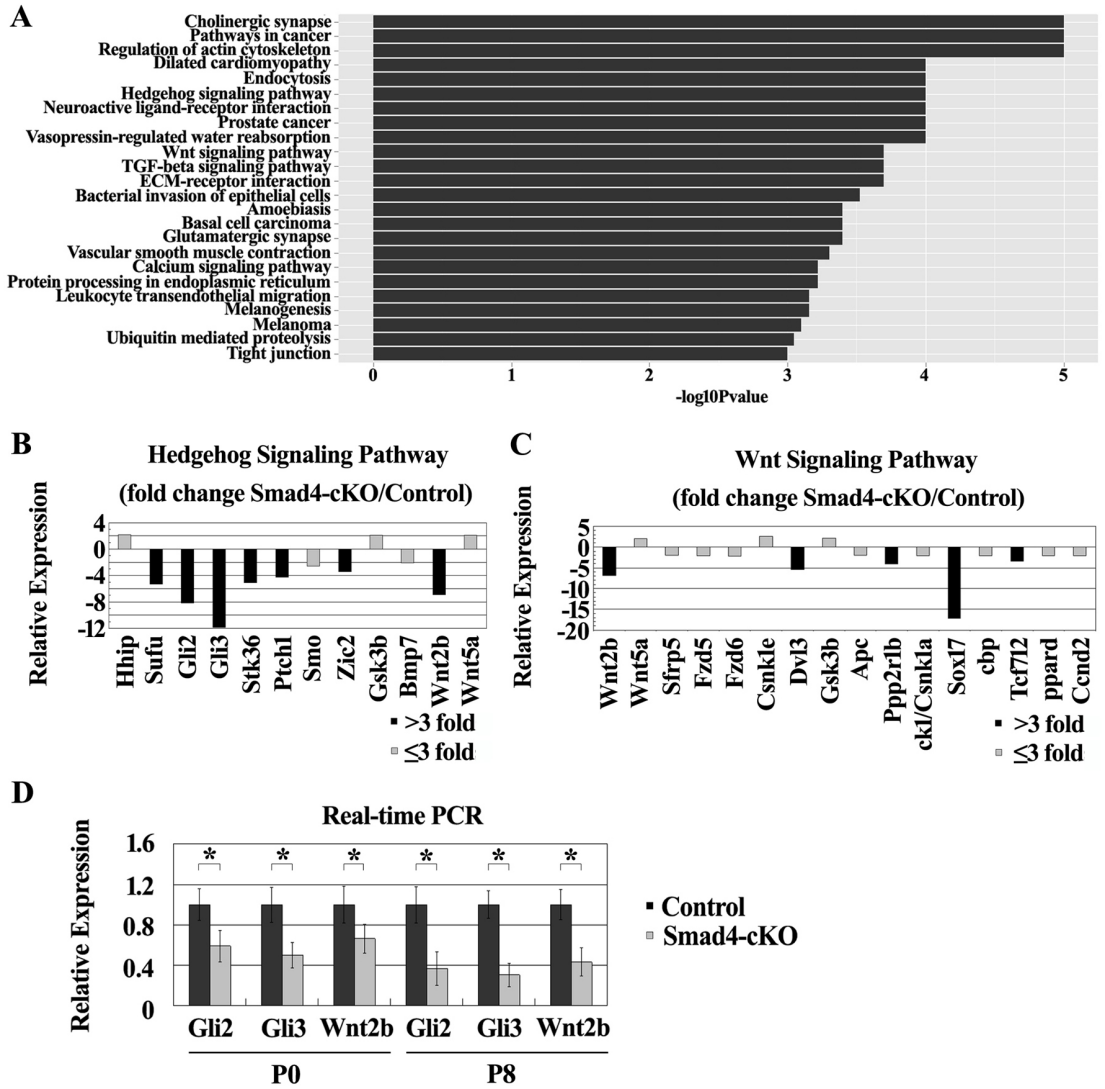
Microarray analysis and Real-time qPCR showed the differentially expressed genes in control and *Smad4*-cKO mice. **(A)** Overview of signaling pathway analysis defined by the SBS Analysis System (http://sas.ebioservice.com/). **(B-C)** Charts showed the expression changes of genes in Hedgehog signaling pathway and Wnt signaling pathway detected by microarray within retina, respectively. Genes with more than 3 fold expression changes were used for further exploration. **(D)** Real-time qPCR was performed to detect the expression of Gli2, Gli3 and Wnt2b within retina at P0 and P8. The expression of Gli2, Gli3 and Wnt2b was down-regulated at P0 and P8. n = 4, *P<0.05. E, embryonic; P, postnatal; M, month.

**Fig 6:**
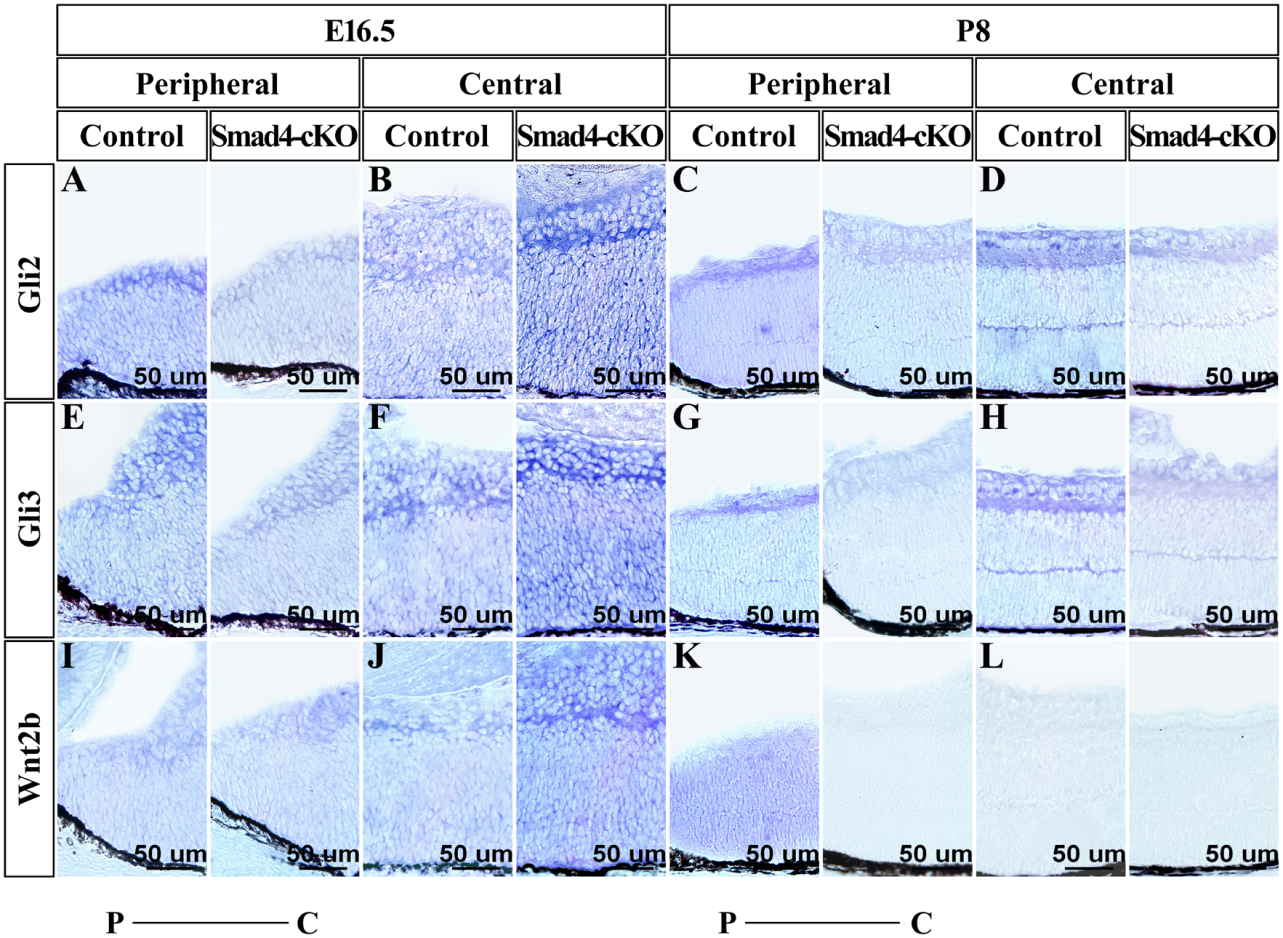
Nonradioactive RNA in situ hybridization was used to confirm Gli2, Gli3 and Wnt2b expression changes in the cKO retinas. **(A-H)** Expression of Gli2 and Gli3 was shown in retina of *Smad4*-cKO mice and control mice at E16.5 and P8. At E16.5, the expression of Gli2 and Gli3 was significantly reduced in the peripheral zone of retina, and slightly up-regulated in the central zone of retina in *Smad4*-cKO mice compared to control. At P8, the expression of Gli2 and Gli3 was distinctly decreased in the peripheral zone and central zone of retina in *Smad4*-cKO mice compared to control. **(I-L)** Expression of Wnt2b was shown in retina of *Smad4*-cKO mice and control mice at E16.5 and P8. The expression of Wnt2b was obviously increased in the central zone of *Smad4*-cKO retina compared to control at E16.5. At P8, the expression of Wnt2b was missing in the peripheral zone of cKO retina. E, embryonic; P, postnatal; M, month.

To understand the molecular mechanism of the retinal phenotype resulted from the *Smad4* deletion in the ocular surface ectoderm, we performed cDNA microarray analyses of the retinas isolated from P0 control and *Smad4*-cKO. 2233 genes were upregulated and 2825 genes were downregulated in the cKO retinas compared to the WT controls using a cutoff of 2-fold. Pathway analysis using the SBS Analysis System (http://sas.ebioservice.com/) identified many signaling pathways that are significantly regulated by *Smad4* (Fig 5A). We focused on two pathways, Hh and Wnt signaling pathways, which have been shown previously to regulate retinal development [22–35]. Most of the regulated genes in these pathways were downregulated in the cKO retina, although a few of them were upregulated. Specifically, *Gli2*, *Gli3*, *Sufu*, *Stk36(Fu)* and *Zic2* are downregulated genes that are positive regulators of the Hh pathway, while *Ptch1* is a downregulated gene that is a negative regulator of Hh pathway but is positively regulated by Hh pathway [36–40] (Fig 5B). *Wnt2b*, *Dvl3*, *Ppp2r1b* and *Tcf712* are down-regulated genes that are positive regulators in the Wnt pathway, while *Sox17* is a downregulated gene that is a negative regulator of Wnt signaling [41–44] (Fig 5C). The expression of a list of the regulated genes was confirmed by quantitative (q) PCR. Gli2, Gli3 and Wnt2b expression was up-regulated at E16.5, and down-regulated at P0 and P8 in cKO retinas compared to the WT controls (Fig 5D, S6B Fig). Nonradioactive RNA in situ hybridization was further used to confirm Gli2, Gli3 and Wnt2b expression changes in the cKO retinas. At E16.5, the expression of Gli2 and Gli3 was significantly reduced in the peripheral zone of retina (Fig 6A and 6E) and slightly up-regulated in the central zone of retina in *Smad4*-cKO mice compared to the WT controls (Fig 6B and 6F). At P8, the expression of Gli2 and Gli3 was noticeably decreased in the peripheral zone and central zone of retina in *Smad4*-cKO mice (Fig 6C, 6D, 6G and 6H). The expression of Wnt2b was noticeably increased in the central zone of retina (Fig 6J), but no significant change was observed in peripheral zone of retina of *Smad4*-cKO mice at E16.5 (Fig 6I). The expression of Wnt2b was not detected in peripheral zone of retina of *Smad4*-cKO mice at P8 (Fig 6K). Taken together, we observed gene expression change in the Hh and Wnt pathways that correlates with the morphological and cell number dynamic changes in the *Smad4*-cKO retinas, suggesting the change of Hh and Wnt pathways may underlie the phenotype in *Smad4*-cKO retinas.

## Discussion

Microphthalmia can be caused by lens dysplasia or failure of retinal differentiation in monogenic mutants [1]. How defects in lens can lead to pathogenesis of microphthalmia remains unclear. We provide evidence that deletion of *Smad4* in the ocular surface ectoderm leads to defective retinal development. Our data suggest an intact ocular surface ectoderm is important for retinal development, and *Smad4* deletion in the surface ectoderm can impact Hh and Wnt signaling in the retina.

Many genes have been known to play key roles in normal ocular development in humans. Among them including *Sox2*, *Otx2*, *Pax6*, have been associated with anophthalmia and microphthalmia in many individuals [1, 2, 13]. We found that specific deletion of *Smad4* in the surface ectoderm leads to microphthalmia, consistent with a recent report [14]. We provide additional evidence that loss of *Smad4* in the surface ectoderm leads to varying degrees of microphthalmia with atresia iridis or even aphakia, and defective formation of ciliary body and iris. In the aphakia mutant, the retina is pleated and corrgated in the center of the eyeball, while in the mutants possessing a lens, the retina forms a cup-structure but smaller than control, indicating that the lens plays an important role in proper orientation of optic vesicles and eye volume maintenance.

We performed detailed analysis of the retinas in the *Smad4*-cKO mice. The retina of the *Smad4*-cKO mice appears thicker and possesses more retinal cells than the retina of the wild-type at the embryonic stages. However, after P3, the retina thickness of Smad4-cKO mice decreases rapidly over time in comparison to control mice. These results are consistent with our proliferation and TUNEL assays. Retinal cells from the cKO mice proliferate more rapidly than those in WT cells before P0, but significantly more apoptosis occurs in the retinal cells from the cKO mice after birth. The changes in retina thickness are likely due to the excessive proliferation early on and the massive apoptosis at later stage.

We also observed dynamic changes in the expression of genes in Hh and Wnt pathways in the retina after *Smad4* deletion in the surface ectoderm. These results are consistent with the documented function of Hh and Wnt signaling in the retina. Previous studies have shown that Hh signaling can activate cell proliferation in developing retina, while its absence results in elevated apoptosis due to activation of p53 pathway [22, 23]. Wnt2b has been shown to be important in maintenance and proliferation of neuroblasts [24]. At E16.5, the expression of Gli2, Gli3 and Wnt2b was higher in the *Smad4*-cKO retina compared to the control. Therefore, at early embryonic stage, the retina of the *Smad4*-cKO mice appears thicker and mutant retinal cells proliferate more rapidly probably due to activation of Wnt and Hh signaling pathway. After birth, the expression of Gli2 and Gli3 was significantly down-regulated throughout the retina in *Smad4*-cKO mice. The expression of Wnt2b was extremely low in both *Smad4*-cKO mice and control mice. Consequently, the massive apoptosis of retinal cells could be caused by inhibition of Hh signaling pathway in *Smad4*-cKO mice after birth. We also observed defective formation of ciliary body and iris at P5, which correlates with a downregulation of Wnt2b in the peripheral zone of retina in *Smad4*-cKO mice after birth. In this regard, canonical Wnt/β-catenin pathway has been shown to play a vital role in inducing ciliary body and iris development from the peripheral retina [25, 26]. Based on these, we hypothesize that *Smad4* deletion in the surface ectoderm leads to defective Hh and Wnt signaling in the retina during development.

The immunostaining results show that the differentiation of retinal cells is also affected in the *Smad4*-cKO mice. From previous research, development of retinal ganglion cells, bipolar cells and Müller glia rely on Hh signaling, and inhibition of Hh signaling pathway results in failed retinal differentiation [27–33]. Retinal rosettes can be observed in the majority of cases of microphthalmia. Inhibition of Hh signaling in explants culture results in retinal rosetting, which can be restored by the addition of Shh protein [34]. Conditional deletion of the Shh gene results in retinal disorganization and retinal resetting [34]. In our results, the ganglion cells of Smad4-cKO retina show rapid differentiation at paracentral zone and central zone, and delayed differentiation was observed at peripheral zone. These are consistent with the changes of Hh genes in these regions at E16.5. The number of ganglion cells exhibited an accelerated increase rate across embryonic stages and a more rapidly reducing rate after birth, probably caused by the excessive proliferation and subsequent apoptosis of retinal cells. The differentiation of bipolar cells may be initiated by Hh signaling and be independent of Hh signaling subsequently, as the differentiation of bipolar cells is delayed but the number of bipolar cells shows rapid increase after P9. The decrease in the number of bipolar cells after P14 is probably caused by excessive apoptosis. The precise mechanism in differentiation of bipolar cells requires further investigation. The differentiation of Müller cells is dependent on Hh signaling [27, 29, 33], thus the down regulation of this pathway observed in cKO mutants is consistent with absence of Müller cells in *Smad4*-cKO retina.

The crosstalk among TGF-β/BMP signaling pathway, Wnt signaling pathway and Hedgehog signaling pathway are implicated in a diverse array of biological processes. Several studies have demonstrated that TGF-β and Wnt ligands can cooperate to regulate differentiation and cell fate [45]. BMP-2 can induce the activation of WNT/β-catenin signaling pathway [46]. Moreover, inhibition of BMP signaling results in activation of Hh signaling pathway in the development of anterior neuroectodermal structures, while activation of BMP signalings negatively regulates Shh transcription during limb development [47, 48]. In incisors teeth, loss of Smad4 in the dental epithelium releases the inhibitory effect of BMP on the SHH pathway and expands the SHH-Gli1 signaling activity [49, 50]. However, in the TGF-β type II receptor gene conditionally deleted embryos, Ihh signal is downregulated in the limb bud mesenchymal cells [51]. In addition, Gli1 can induce the expression of Wnt2b, Wnt4 and Wnt7b, and Wnt activity is necessary to activate ectopic Gli3 expression [52, 53]. Therefore, if there are the signal molecules released from the surface ectoderm in *Smad4*-cKO mice, they could be TGF-β, Hh or Wnt family members. Future research is needed to determine whether there are signal molecules regulated by *Smad4* in the ocular surface ectoderm that can regulate Hh and wnt signaling in the retina, or the structural changes in the lens can lead to dysregulated Hh and wnt signaling and defective retinal development. This is important because in patients with microphthalmia, apoptosis of retinal cells, failure of retinal differentiation and retinal detachment are the main causes of blindness. Based on our study, microphthalmia patients may have cataract as their initial symptoms, and harmful signaling molecules may be released from these defective lenses, or the defective lens structure may result in abnormal changes in the fundus. Based on our study, activation of Hh signaling may be used to protect retinal cells, activate retinal differentiation and reduce the formation retinal rosettes, while manipulating Wnt signaling could treat the dysplasia of ciliary body and iris. Since the formation of ciliary body and iris has been completed before birth in human beings, the timing of treatment may be critical for treating microphthalmia patients.

In conclusion, our present work provides evidence of pathological retinal change in a microphthalmia mouse model, and proposes that the pathological change of retina in microphthalmia is associated with dysregulated Hh and Wnt signaling in the retinas. Manipulating Wnt and/or Hh signaling may have implications in treating retinal phenotypes associated with microphthalmia.

## Supporting Information

S1 Fig Ocular dysplasia in *Smad4*-cKO mutants compared to the wild type mice. **(A-C)** Representative images of *Smad4*-cKO and WT mouse at 2 month.
(TIF)

S2 Fig Varying degrees of microphthalmia were observed in *Smad4*-cKO mutants. **(A-C, E-G)** Pictures showed the ocular dysplasia in the *Smad4*-cKO compared to the WT mice at embryonic stages. At E10.5, in both WT and cKO mice, the optic vesicle and the thickened surface ectoderm invaginated together to form the optic cup, and no difference in the size of the optic cup was observed. At E12.5, the mutant eye was slightly smaller, and then the volume of the mutant eye became significantly smaller compared to the WT controls as the embryos developed. Moreover, the cKO mice showed small lens and congenital cataracts, and a nodule of dysplastic primary vitreous accompanied by large numbers of vessels and nucleated erythrocytes attached to the posterior lens capsule (arrows). **(D, H, I)** Huge retinal fold, retinal rosettes and retinal detachment presented in *Smad4*-cKO mice at 1M. **(I)** is the enlargement of boxed area of **(H)**. In the retinal rosettes (*), the nuclei polarized away from a “lumen”. **(J)** Measurement of eyeball size was performed in antero-posterior diameter and right-left diameter (μm). The size of the mutant eye became definitely smaller than that of normal mouse as the embryo growing, as indicated by decreased length of both antero-posterior diameter and left-right diameter. n = 9. *P<0.01. E, embryonic; P, postnatal; M, month; Lp, lens placode; Pnr, presumptive neural retina; L, lens; C, cornea; R, retina.
(TIF)

S3 Fig Deletion of *Smad4* led to changes of retina thickness. **(A)** Charts indicated the variation trend of retina thickness (μm) from E12.5 to 1M at nasal central zone, paracentral and peripheral zone. From E12.5, the cKO retina appeared thicker compared to control retina at central zone and paracentral zone before birth. After P3, the thickness of the cKO retina reduced dramatically and uniformly at central zone, paracentral zone and peripheral zone. n = 9, *P<0.05. **(B)** Charts indicated the total number of retinal cells per retinal section from E12.5 to 1M. The total number of retinal cells in *Smad4* defective embryo was significantly more than that of control. After P3, the total number of cKO retinal cells sharply declined. n = 9, *P<0.05. **(C)** Charts indicated the variation trend of ganglion cell layer thickness (μm) from E16.5 to P5 at nasal central zone, paracentral and peripheral zone. In the *Smad4*-cKO, the ganglion cells layer showed delayed differentiation at peripheral zone at E16.5. n = 9, *P<0.05. **(D)** Charts indicated the variation trend of neuroblast layer thickness (μm) from E16.5 to P5 at nasal central zone, paracentral and peripheral zone. n = 9, *P<0.05. **(E, F)** Charts indicated the variation trend of inner nuclear layer thickness (μm) and outer nuclear layer thickness (μm) from P7 to 1M at nasal central zone, paracentral and peripheral zone, respectively. In *Smad4*-cKO, the thickness of inner nuclear layer and outer nuclear layer reduced dramatically and uniformly at central zone, paracentral zone and peripheral zone. n = 9, *P<0.05. P, postnatal; M, month.
(TIF)

S4 Fig Cell proliferation and cell death in the Smad4-cKO retina. **(A)** Total proliferation rate was calculated with BrDU positive cells per retinal section divided by total number of retinal cells per retinal section. n = 9, No significant difference between the two groups. **(B)** Number of BrDU positive cells was shown in *Smad4*-cKO mice and control mice at central zone, paracentral zone and peripheral zone in nasal and temporal side, respectively, at E14.5. n = 9, *P<0.05. **(C)** Proliferation rate was calculated with BrDU positive cells per area divided by total number of retinal cells per area in *Smad4*-cKO mice and control mice, at central zone, paracentral zone, peripheral zone in nasal and temporal side, respectively, at E14.5. n = 9, No significant difference between the two groups. **(D)** Number of apoptotic cells was shown in *Smad4*-cKO mice and control mice at central zone, paracentral zone and peripheral zone in nasal and temporal side, respectively, at P9. The *Smad4*-deficient retina exhibited grossly more apoptosis than the control especially at peripheral zone. n = 9, **P<0.01. E, embryonic; P, postnatal.
(TIF)

S5 Fig Loss of *Smad4* affected the differentiation of retinal cells. **(A)** Immunostaining was performed to label retinal ganglion cells (red) in *Smad4*-cKO and control mice at the nasal side of E16.5. Delayed differentiation of ganglion cells presented at the nasal peripheral zone in *Smad4*-cKO (white arrows). **(B)** The number of retinal ganglion cells was shown in *Smad4*-cKO and control mice. In the WT retina, the number of retinal ganglion cells presented a progressive increase across embryonic stages and a subsequent, substantial reduction due to retinal remodeling during the first postnatal week. However, in the cKO retina, the total number of ganglion cells was apparently more than that of control across embryonic stages, and decreased more significantly after P3 n = 9, *P<0.05. **(C)** The number of retinal bipolar cells was shown in *Smad4*-cKO and control mice. In the cKO retina, the bipolar cells showed delayed differentiation and the total number of bipolar cells was obviously less at P9. n = 9, *P<0.05. **(D)** The number of retinal ganglion cells was shown in *Smad4*-cKO and control mice at nasal central zone, paracentral zone, peripheral zone, as well as temporal central zone, paracentral zone, peripheral zone of E16.5. Delayed differentiation of ganglion cells was shown at peripheral zone in *Smad4*-cKO. n = 9, *P<0.05. **(E)** The number of retinal bipolar cells was shown in *Smad4*-cKO and control mice at nasal central zone, paracentral zone, peripheral zone, as well as temporal central zone, paracentral zone, peripheral zone of P9. In cKO retina, the bipolar cells showed delayed differentiation mainly at peripheral zone. n = 9, *P<0.05. E, embryonic; P, postnatal; M, month; GCL, ganglion cell layer; NBL, neuroblastic layer.
(TIF)

S6 Fig Microarray analysis and Real-time qPCR showed the differentially expressed genes in control and *Smad4*-cKO mice. **(A)** Charts showed the expression changes of genes in TGF β signaling pathway detected by microarray within retina. **(B)** Real-time qPCR was performed to detect the expression of Gli2, Gli3 and Wnt2b within retina at E16.5. n = 4, *P<0.05. E, embryonic.
(TIF)

S1 Table Primary antibodies and secondary antibodies. (DOC)

S2 Table Primary sequences used for real-time PCR and in situ hybridization. (DOC)
