# Supplementary figures and images for: Requirement of Smad4 from Ocular Surface Ectoderm for Retinal Development
Source: PLoS One. 2016 Aug 5;11(8):e0159639. doi: 10.1371/journal.pone.0159639 (PMC4975478; doi:10.1371/journal.pone.0159639)

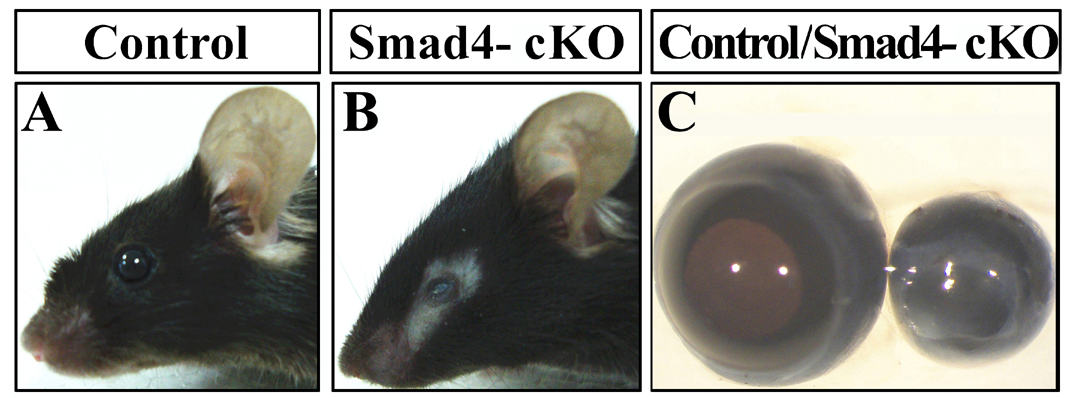

Supplement: S1 Fig — (A-C) Representative images of Smad4-cKO and WT mouse at 2 month. (TIF) [file pone.0159639.s001.tif]

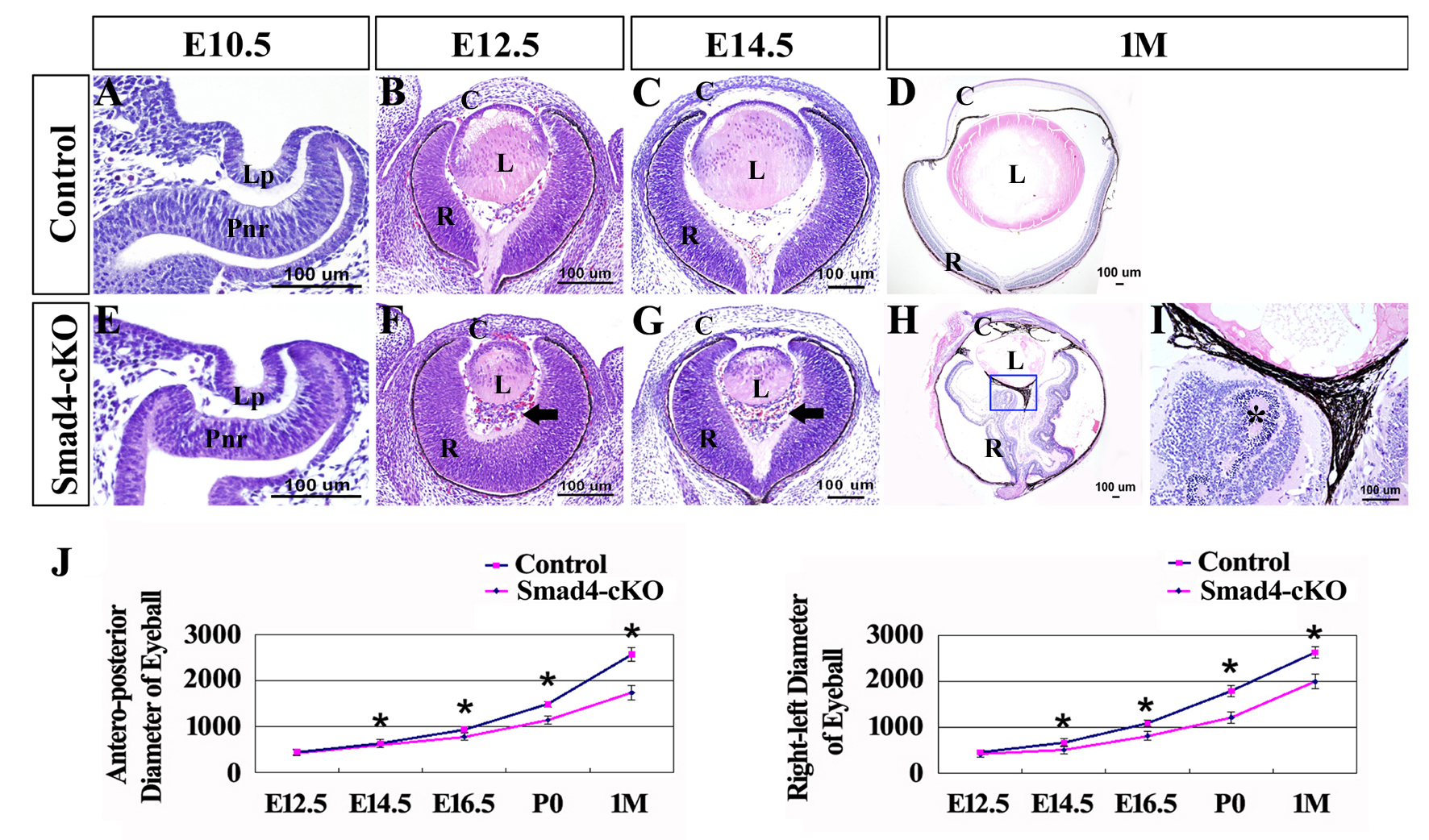

Supplement: S2 Fig — (A-C, E-G) Pictures showed the ocular dysplasia in the Smad4-cKO compared to the WT mice at embryonic stages. At E10.5, in both WT and cKO mice, the optic vesicle and the thickened surface ectoderm invaginated together to form the optic cup, and no difference in the size of the optic cup was observed. At E12.5, the mutant eye was slightly smaller, and then the volume of the mutant eye became significantly smaller compared to the WT controls as the embryos developed. Moreover, the cKO mice showed small lens and congenital cataracts, and a nodule of dysplastic primary vitreous accompanied by large numbers of vessels and nucleated erythrocytes attached to the posterior lens capsule (arrows). (D, H, I) Huge retinal fold, retinal rosettes and retinal detachment presented in Smad4-cKO mice at 1M. (I) is the enlargement of boxed area of (H). In the retinal rosettes (*), the nuclei polarized away from a “lumen”. (J) Measurement of eyeball size was performed in antero-posterior diameter and right-left diameter (μm). The size of the mutant eye became definitely smaller than that of normal mouse as the embryo growing, as indicated by decreased length of both antero-posterior diameter and left-right diameter. n = 9. *P<0.01. E, embryonic; P, postnatal; M, month; Lp, lens placode; Pnr, presumptive neural retina; L, lens; C, cornea; R, retina. (TIF) [file pone.0159639.s002.tif]

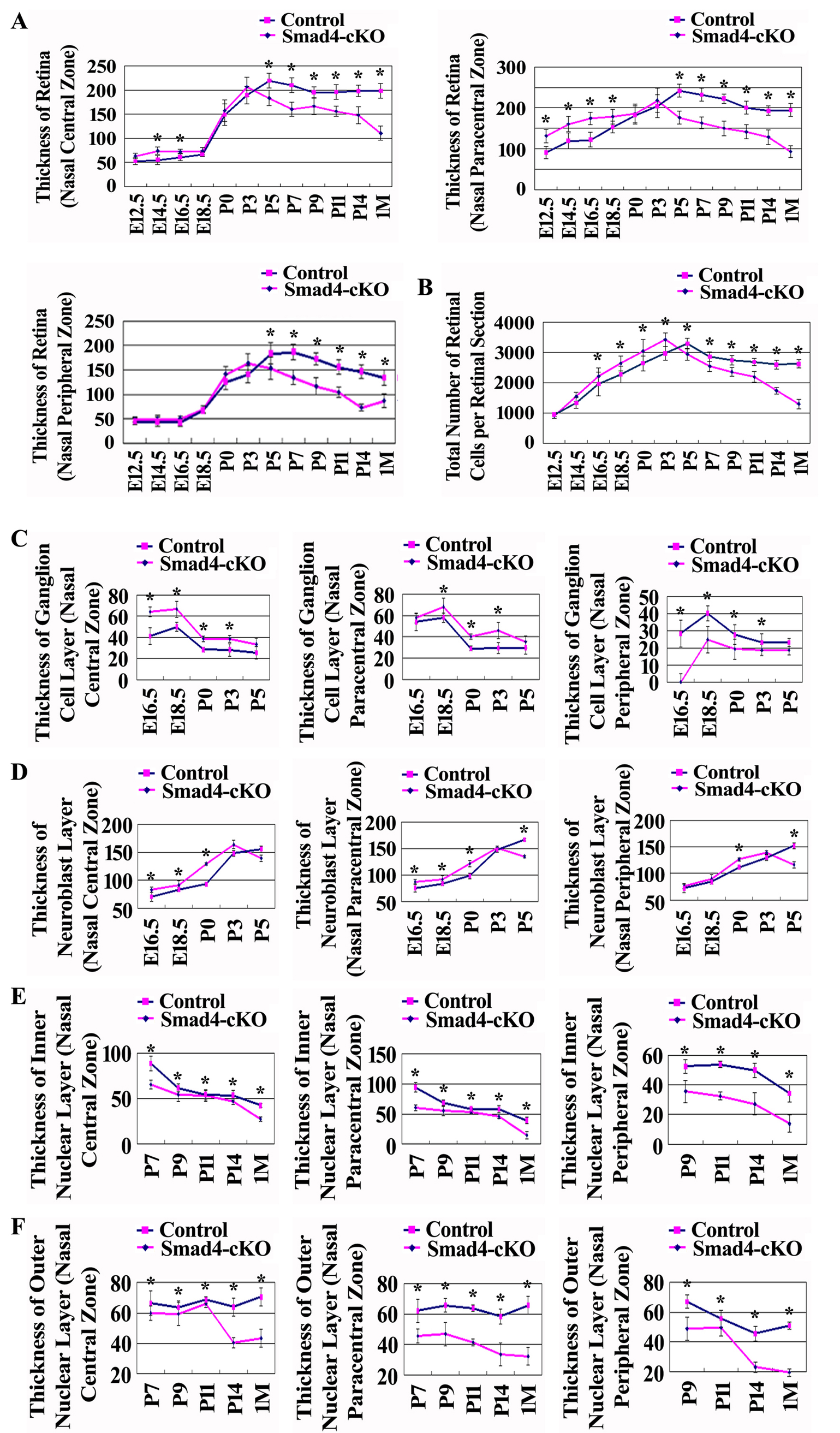

Supplement: S3 Fig — (A) Charts indicated the variation trend of retina thickness (μm) from E12.5 to 1M at nasal central zone, paracentral and peripheral zone. From E12.5, the cKO retina appeared thicker compared to control retina at central zone and paracentral zone before birth. After P3, the thickness of the cKO retina reduced dramatically and uniformly at central zone, paracentral zone and peripheral zone. n = 9, *P<0.05. (B) Charts indicated the total number of retinal cells per retinal section from E12.5 to 1M. The total number of retinal cells in Smad4 defective embryo was significantly more than that of control. After P3, the total number of cKO retinal cells sharply declined. n = 9, *P<0.05. (C) Charts indicated the variation trend of ganglion cell layer thickness (μm) from E16.5 to P5 at nasal central zone, paracentral and peripheral zone. In the Smad4-cKO, the ganglion cells layer showed delayed differentiation at peripheral zone at E16.5. n = 9, *P<0.05. (D) Charts indicated the variation trend of neuroblast layer thickness (μm) from E16.5 to P5 at nasal central zone, paracentral and peripheral zone. n = 9, *P<0.05. (E, F) Charts indicated the variation trend of inner nuclear layer thickness (μm) and outer nuclear layer thickness (μm) from P7 to 1M at nasal central zone, paracentral and peripheral zone, respectively. In Smad4-cKO, the thickness of inner nuclear layer and outer nuclear layer reduced dramatically and uniformly at central zone, paracentral zone and peripheral zone. n = 9, *P<0.05. P, postnatal; M, month. (TIF) [file pone.0159639.s003.tif]

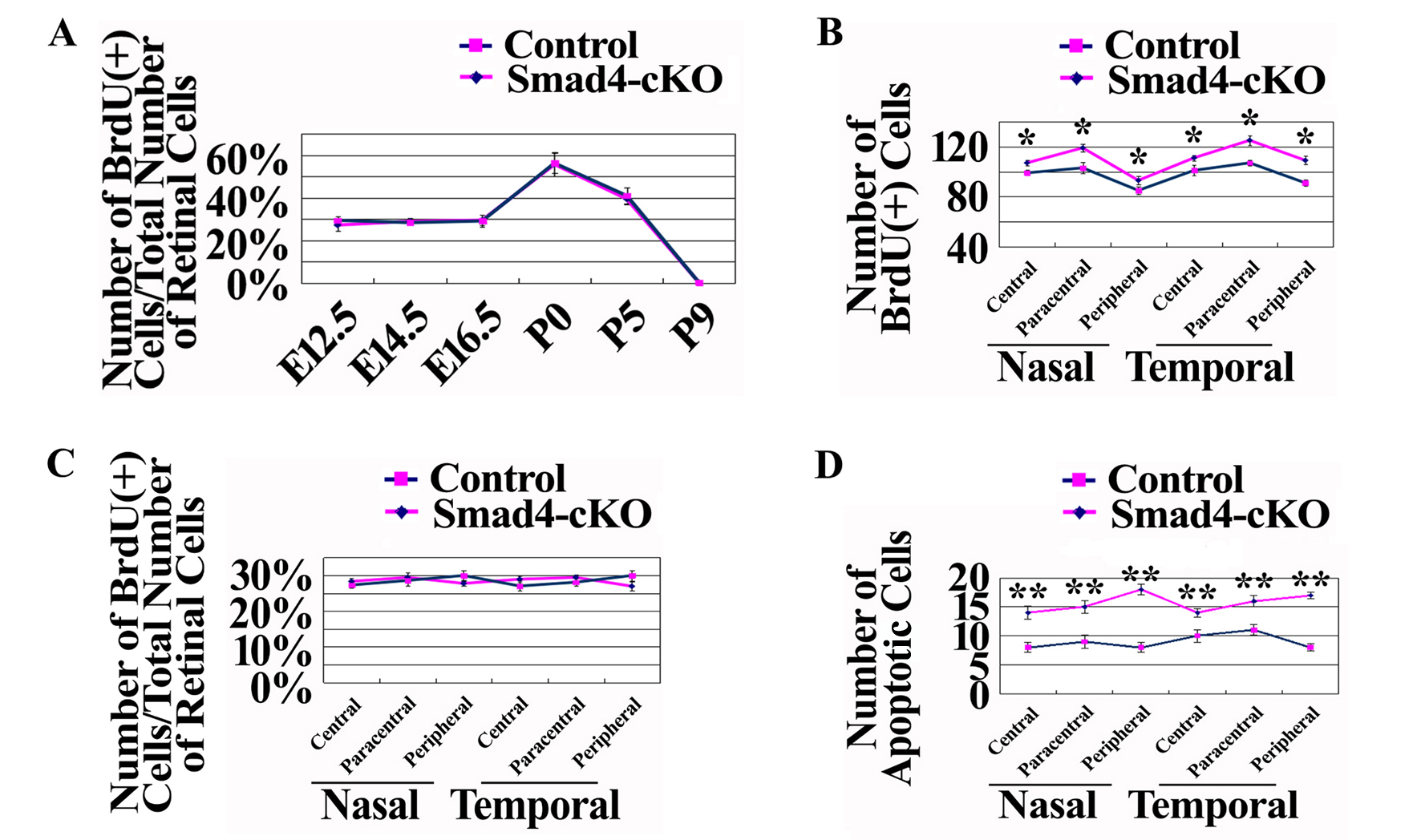

Supplement: S4 Fig — (A) Total proliferation rate was calculated with BrDU positive cells per retinal section divided by total number of retinal cells per retinal section. n = 9, No significant difference between the two groups. (B) Number of BrDU positive cells was shown in Smad4-cKO mice and control mice at central zone, paracentral zone and peripheral zone in nasal and temporal side, respectively, at E14.5. n = 9, *P<0.05. (C) Proliferation rate was calculated with BrDU positive cells per area divided by total number of retinal cells per area in Smad4-cKO mice and control mice, at central zone, paracentral zone, peripheral zone in nasal and temporal side, respectively, at E14.5. n = 9, No significant difference between the two groups. (D) Number of apoptotic cells was shown in Smad4-cKO mice and control mice at central zone, paracentral zone and peripheral zone in nasal and temporal side, respectively, at P9. The Smad4-deficient retina exhibited grossly more apoptosis than the control especially at peripheral zone. n = 9, **P<0.01. E, embryonic; P, postnatal. (TIF) [file pone.0159639.s004.tif]

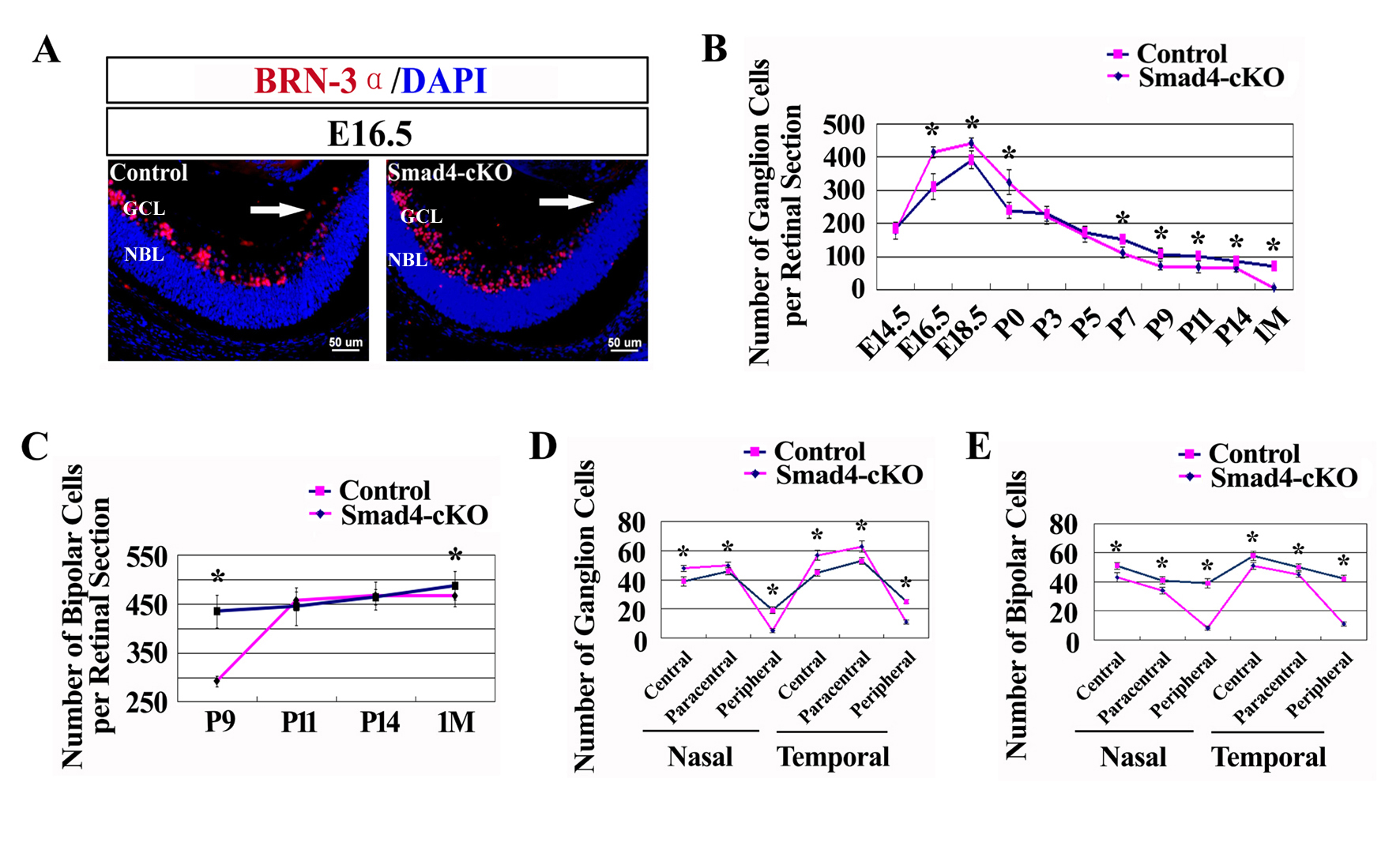

Supplement: S5 Fig — (A) Immunostaining was performed to label retinal ganglion cells (red) in Smad4-cKO and control mice at the nasal side of E16.5. Delayed differentiation of ganglion cells presented at the nasal peripheral zone in Smad4-cKO (white arrows). (B) The number of retinal ganglion cells was shown in Smad4-cKO and control mice. In the WT retina, the number of retinal ganglion cells presented a progressive increase across embryonic stages and a subsequent, substantial reduction due to retinal remodeling during the first postnatal week. However, in the cKO retina, the total number of ganglion cells was apparently more than that of control across embryonic stages, and decreased more significantly after P3 n = 9, *P<0.05. (C) The number of retinal bipolar cells was shown in Smad4-cKO and control mice. In the cKO retina, the bipolar cells showed delayed differentiation and the total number of bipolar cells was obviously less at P9. n = 9, *P<0.05. (D) The number of retinal ganglion cells was shown in Smad4-cKO and control mice at nasal central zone, paracentral zone, peripheral zone, as well as temporal central zone, paracentral zone, peripheral zone of E16.5. Delayed differentiation of ganglion cells was shown at peripheral zone in Smad4-cKO. n = 9, *P<0.05. (E) The number of retinal bipolar cells was shown in Smad4-cKO and control mice at nasal central zone, paracentral zone, peripheral zone, as well as temporal central zone, paracentral zone, peripheral zone of P9. In cKO retina, the bipolar cells showed delayed differentiation mainly at peripheral zone. n = 9, *P<0.05. E, embryonic; P, postnatal; M, month; GCL, ganglion cell layer; NBL, neuroblastic layer. (TIF) [file pone.0159639.s005.tif]

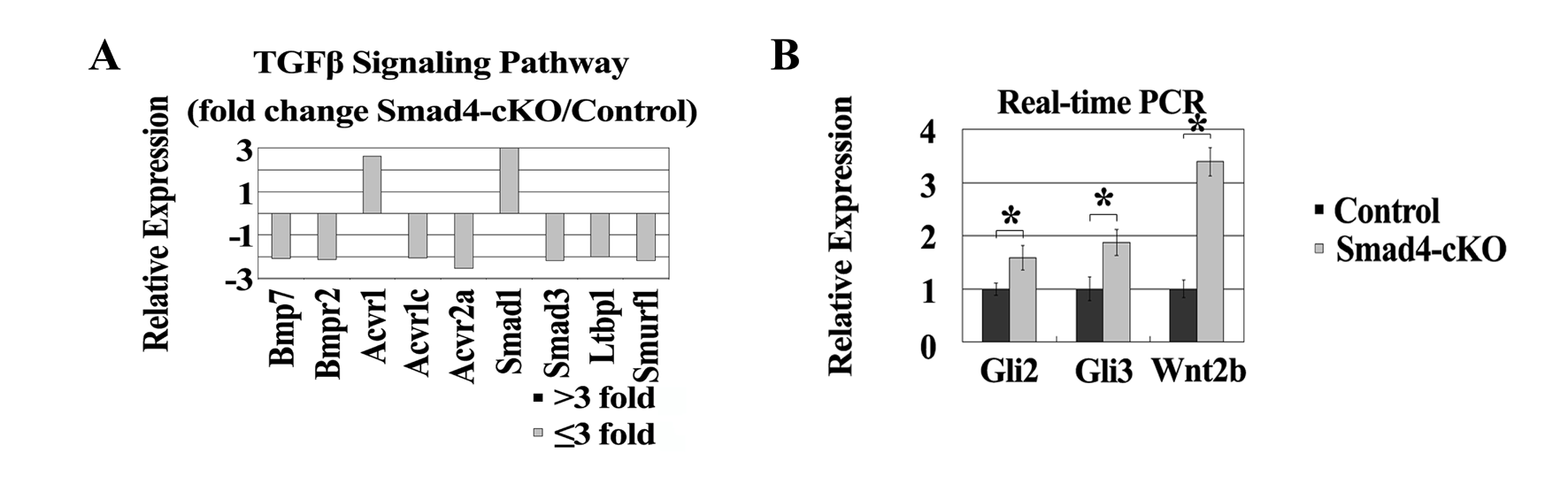

Supplement: S6 Fig — (A) Charts showed the expression changes of genes in TGF β signaling pathway detected by microarray within retina. (B) Real-time qPCR was performed to detect the expression of Gli2, Gli3 and Wnt2b within retina at E16.5. n = 4, *P<0.05. E, embryonic. (TIF) [file pone.0159639.s006.tif]
