# Supplementary material for: Requirement of Smad4 from Ocular Surface Ectoderm for Retinal Development
Source: PLoS One. 2016 Aug 5;11(8):e0159639. doi: 10.1371/journal.pone.0159639 (PMC4975478; doi:10.1371/journal.pone.0159639)
Supplement: S1 Table — (DOC) [file pone.0159639.s007.doc]

**S1 Table.** Primary antibodies and secondary antibodies

| **Antibody** | Host | Dilution | Source |
| --- | --- | --- | --- |
| Smad4 | Goat | 1:100 | Santa Cruz, sc-1909 |
| Brn-3α | Goat | 1:200 | Santa Cruz, sc-31984 |
| PKC α | Rabbit | 1:200 | Santa Cruz, sc-208 |
| GFAP | Rabbit | 1:500 | Abcam, ab7260 |
| BrdU | Mouse | 1:200 | Sigma, B8434 |
| Alexa 555-conjugated anti-goat IgG | Donkey | 1:1000 | Life Technologies, A-21432 |
| Alexa 488-conjugated anti-rabbit IgG | Donkey | 1:1000 | Life Technologies, A-21206 |
| Alexa 555-conjugated anti-rabbit IgG | Donkey | 1:1000 | Life Technologies, A-31572 |
| Alexa 555-conjugated anti-mouse IgG | Donkey | 1:1000 | Life Technologies, A-31570 |
