# Supplementary material for: Requirement of Smad4 from Ocular Surface Ectoderm for Retinal Development
Source: PLoS One. 2016 Aug 5;11(8):e0159639. doi: 10.1371/journal.pone.0159639 (PMC4975478; doi:10.1371/journal.pone.0159639)
Supplement: S2 Table — (DOC) [file pone.0159639.s008.doc]

**S2 Table.** Primary sequences used for real-time PCR and in situ hybridization

| **Genes** | Sense(5’- 3’) | Antisense(5’- 3’) |
| --- | --- | --- |
| Gli2 | GCCAAGCCATGGTCACATCA | CTGTGTCCTGAGATGGCTGA |
| Gli3 | CTCACCTGATTGGGATGTGTCT | TGGAAGACAGTTCCTCCCCTA |
| Wnt2b | CCGAGGTGGCAAACATCCTA | TCGTGGAACGTGCAGTAGTT |
| Sp6-Gli2-T7 | ATTTAGGTGACACTATAGGCCAAGCCATGGTCACATCA | TAATACGACTCACTATAGGGCTGTGTCCTGAGATGGCTGA |
| Sp6-Gli3-T7 | ATTTAGGTGACACTATAGCTCACCTGATTGGGATGTGTCT | TAATACGACTCACTATAGGGTGGAAGACAGTTCCTCCCCTA |
| Sp6-Wnt2b-T7 | ATTTAGGTGACACTATAGCCGAGGTGGCAAACATCCTA | TAATACGACTCACTATAGGGTCGTGGAACGTGCAGTAGTT |
